# Parenthood in transition – Somali-born parents’ experiences of and needs for parenting support programmes

**DOI:** 10.1186/s12914-016-0082-2

**Published:** 2016-02-16

**Authors:** Fatumo Osman, Marie Klingberg-Allvin, Renée Flacking, Ulla-Karin Schön

**Affiliations:** Department of Women’s and Children’s Health, Karolinska Institutet, Stockholm, Sweden; School of Education, Health and Social Studies, Dalarna University, Falun, Sweden

**Keywords:** Immigrant, Mental health, Migration, Parenting, Parenthood in transition, Qualitative method, Somali parents

## Abstract

**Background:**

Pre- and post-migration trauma due to forced migration may impact negatively on parents’ ability to care for their children. Little qualitative work has examined Somali-born refugees’ experiences. The aim of this study is to explore Somali-born refugees’ experiences and challenges of being parents in Sweden, and the support they need in their parenting.

**Methods:**

A qualitative descriptive study was undertaken. Data were collected from four focus group discussions (FGDs) among 23 Somali-born mothers and fathers living in a county in central Sweden. Qualitative content analysis has been applied.

**Results:**

A main category, *Parenthood in Transition*, emerged as a description of a process of parenthood in transition. Two generic categories were identified: *Challenges*, and *Improved parenting. Challenges* emerged from leaving the home country and being new and feeling alienated in the new country. In *Improved parenting*, an awareness of opportunities in the new country and ways to improve their parenting was described, which includes how to improve their communication and relationship with their children. The parents described a need for information on how to culturally adapt their parenting and obtain support from the authorities.

**Conclusions:**

Parents experienced a process of parenthood in transition. They were looking to the future and for ways to improve their parenting. Schools and social services can overcome barriers that prevent lack of knowledge about the new country’s systems related to parenthood. Leaving the home country often means separation from the family and losing the social network. We suggest that staff in schools and social services offer parent training classes for these parents throughout their children’s childhood, with benefits for the child and family.

## Background

Previous studies have stressed the obstacles that Somali families encounter when leaving their home country and family behind due to conflict and war, and coming to receiving countries as a refugees. These challenges may affect how parents manage their role as parents [1–3]. In order to offer adequate parenting support for Somali-born parents and their children who fled from war and conflict, a good understanding of their experiences and opportunities for support is crucial to investigate. However studies that address Somali-born parents’ specific needs of parenting support in Swedish context are limited.

The civil war in Somalia started at the beginning of 1991. Since then, many Somalis have been forcedly migrated and displaced in and outside the country [4]. Forced migration in this context thus regard non-valuntary migration from war and conflict [5]. In recent years the Somali population in Sweden have increased and are today one of the fastest growing group among ethnic minorities in Sweden [6]. Pre-migration life for immigrants from war-torn and conflict areas is associated with experience of trauma, violence and loss of family members [7–9]. Such experiences and immigration to a new social/cultural context are often challenging, and migrants may experience loss of their homeland, loneliness and lack of cultural and social support, as well as unemployment, acculturation difficulties, stigma and discrimination in the host country. This in turn creates poorer mental health and difficulties for immigrants to integrate into society in the host country [8–14]. Despite the fact that pre-migration trauma has an impact on immigrants’ mental health, as described in Somali refugees [15–17], the stressful life in the new country, including poverty, unemployment, social isolation and discrimination, has been reported to have an even stronger effect on mental distress, and is termed “post-migration trauma”. Poor mental health in parents and poor self-efficacy in parenting [18] have been shown to be associated with poor child/adolescent health, education, employment and criminality outcome [18].

### Immigrant family transition to a new country

Several international studies both qualitative and quantitative have shown that the daily stressors and adjusting as a parent in a new country will render difficulties generally for immigrant parents in a new culture [19–22]. For instance, qualitative studies conducted on Somali families in Finland and North America reported lack of community and social support which creates isolation for parents. Furthermore, lack of information about parenting systems as well as parents’ rights in the host country is an obstacle that parents meet in the new country. Fear of the children’s independence and fear of the social services is correlated to a feeling of powerlessness and a lack of guidance for their children in the new country [1–3, 19, 23].

As a result of immigration, Somali families face many challenges regarding parenting in their new home country. Children are integrated into the new society faster than their parents and hence parents face a struggle relating to how to be a role model for their children [24]. The parents’ parenting style, especially if authoritarian, is frequently questioned by the Somali children, and parents needed to negotiate and/or adapt [1, 3, 25]. An authoritarian parenting style has been described as parents trying to shape and control their children’s attitudes and behaviour [26]. Experiences of compromised roles when migrating have also been described to exist between husband and wife in Somali and other immigrant families. Traditionally in Somalia, the father was the disciplinarian and the mother the nurturer, but in the new environment this changes to a more equal relationship [1–3, 21]. Some studies report that these changing roles have advantages and disadvantages. They create more burden for Somali mothers; also, the daily stressors may cause marital problems and even lead to divorce [2]. On the other hand, some Somali fathers become more involved in the family [19].

As a part of the transition, recent immigrant families with a residence permit in Sweden and with insufficient income are automatically offered social support in terms of social assistance. Thus, the Swedish welfare system is for the whole population, not only for the immigrants or for vulnerable individuals [27]. Social welfare includes monthly child benefits for all parents, subsidized day-care, free school and housing support for people with low income [28].

### A framework for understanding immigrant parenting

A framework for understanding immigrant parenting has been developed by J Ochocka and R Janzen [29] and used by several authors [21, 30]. According to Ochocka and Janzen [29], the process of adapting to a new country and a new way of parenting has been explained as a dynamic process affected by parents’ previous parenting styles and cultural parenting orientations. The authors have highlighted different components within the framework that affect parenting as well as contribute to transition in parenthood. These components start with the *cultural parenting orientation*, which is about the cultural beliefs and family values that parents have formed, shaped by religion and culture, as well as the expectations they have of their children, which are related to their family beliefs and values. The *parenting styles* are the second component, which describes how parents interact with their children in relation to their cultural parenting orientation. The third component is the *host country context*, in which parents start to explore similarities and differences between the cultural parenting orientation and parenting style they have been brought up with, and norms in the host country. The fourth component is *modifications of orientations and styles*, which describes how parents start to adjust and modify their parenting in the new country. The fifth component, *parenting contribution*, comprises the process of making adjustments to the host country. This is not a one-way process; immigrant parents adapt to their new home country as well as contribute to the society they are living in. Finally, the sixth component encompasses *parenting support*, which is the parenting support parents need to have in order to understand and adjust to the new host country. Parenting support facilitates the process of integration between immigrants and the local population.

The struggle of being a parent is universal. For parents who have experience of forced migration and/or are struggling to adapt to the new host country, this struggle is greater. Evidence suggests that giving support to parents in their parenting role has an impact on children’s mental health outcome. For this reason, municipalities in Sweden offer their residents a structured parenting programme both individual and in groups. However, these parenting programmes do not reach all residents, especially those from ethnic minorities [31]. To be able to give parenting support to Somali-born parents, we need to explore what kind of support they need and how their experience of being a parent in the host country affects their parenting. Since the research that explores the specific need for parenting support of Somali-born parents in a new context is limited, a deeper knowledge of the subject is needed to give insight into how to develop culturally sensitive parenting support programmes [1, 3]. From a public health perspective this knowledge is important to decrease health differences in society.

This study is a part of a randomized controlled trial (RCT) on developing and evaluating a parenting support programme for Somali-born parents living in Sweden. The purpose of the present study is to explore Somali-born refugees’ experiences and challenges of being parents in Sweden, and the support they need in their parenting.

## Methods

This study employed a qualitative approach using focus group discussions (FGDs) [32]. Using FGDs gives us insight into participants’ perspectives of a particular phenomenon [32]. The FGD method was used in order to gain an understanding of participants’ perspectives of being parents in the new host country and of their need for support.

The criterion for qualitative research review guidelines - RATS in BioMed Central (BMC) has been followed [33]. According to the guideline the manuscript should be included the reason for choosing the qualitative method and step by step how the procedure of sampling, recruitment, data collection, ethics and data analysis has been conducted.

### Participants and recruitment strategy

Purposive sampling of Somali-born parents living in a municipality in central Sweden was applied. The inclusion criteria for this study were: Somali-born parents who have been living in Sweden with their child for a minimum of one year.

Contact was made with the Somali associations and with Somali key persons in the municipality to gain access to Somalis living in the municipality. The Somali associations and key persons invited Somali-born parents to an information meeting about the study. The information meeting was arranged at one of the Somali associations’ venues. Approximately 30–40 Somali people attended the meeting. Information about the study purpose, procedures and how the results would be presented was given both in writing and orally in Somali language by the first author (F.O.) who is originally from Somalia. Those interested left their telephone number to be contacted later by F.O. Sixteen people gave their consent to participate, twelve women and four men. An additional ten participants were recruited through snowballing [34]. The participants were given the opportunity to choose what kind of FGD they wanted to participate in: a mixed group consisting of both mothers and fathers or a non-mixed group. Written informed consent was collected from the participants.

Three parents, two mothers and one father, who had consented to participate were unable to join the FGD because of sickness or other personal reasons. A total of 23 mothers and fathers participated in four FGDs. The age range for the mothers was 22–48 years old, and for fathers 26–53 years old. They had lived in Sweden for 3 to18 years (for mothers) and 4 to 10 years (for fathers), they had been together with their children between 1 and 18 years (for mothers) and between 2 and 7 years (for fathers). The characteristics of the participants are presented in Table 1.

**Table 1:** Demographic characteristics of the participants

	Mothers = 15 (65 %)	Fathers = 8 (35 %)
Age	22–48 years (M^a^ = 34.6 years)	26–53 years (M = 49 years)
Time in Sweden	3–18 years (M = 5.7 years)	4–10 years (M = 5.6 years)
Time together with the children	1–18 years (M = 5 years)	2–7 years (M = 4 years)
Number of children	1–6 children (M = 3.7)	1–13 children (M = 7.4)
Marital status	11 married,	8 married
	3 divorced	
	1 widowed	
Employment status	3 working	3 working
	4 studying	1 studying
	3 unemployed	2 unemployed
	5 on parental leave	1 job training
		1 on parental leave

^a^M = median

### Data collection

A pre-defined thematic guideline was constructed and pilot tested with a mixed group of Somali parents. All focus groups were asked the same initial questions: “Can you describe your experience of being a parent in Sweden compared with Somalia?”, “How has the migration and experience of war affected you as a parent and your child?” and “What kind of support related to your parenting do you need?” Additional follow up questions were asked based on the guideline. Two FGD were gender mixed, one for mothers and one for fathers. All four FGDs were carried out in Somali language. Three FGDs were conducted by the first author (F.O.) and observed by a Somali-speaking female observer. The FGD for the “father group” was moderated and observed by two Somali-speaking male facilitators. The moderator’s role was to present the subject and ensure that the participants kept to the subject. The observer’s role was to take notes during the discussion about the group interaction. After each FGD the moderator and observer discussed the outcome and compared notes.

All FGDs were tape-recorded and lasted between 1 and 1½ hours.

### Data analysis

Qualitative content analysis was used to analyse the data [35]. All interview data were transcribed verbatim and translated from Somali into English (45 700 words in English) by a professional translator. All interviews were also listened to by the first author (F.O.) for accuracy and to take notes. In order to gain a sense of content understanding, the analysis started with reading all transcribed data inductively several times. Then, data were read word by word to derive codes by highlighting words from the text that appeared to capture key thoughts or concepts. During this process the first author made notes of thoughts and ideas. This open coding was extracted onto coding sheets to further organise and group data. From this step all data were handled in a joint document and sorted into categories based on their similarities and differences. These emergent categories were used to organize and group codes into clusters [35]. Definitions for each category, sub-category and code were developed and the relationship between them was defined based on their “belonging together” [35], concurrence, antecedents or consequences [36]. This progress was discussed by the first author, F.O., and the last author, U.-K.S. The analysis was repeated back and forth. Initially, 16 sub-categories emerged from the analyses. After further discussions and abstractions between all the authors, a total of six sub-categories were identified and agreed on. The main category, *Parenthood in transition,* emerged from the two generic categories, *Challenges* and *Improved parenting*. The categories that emerged from the different FGDs did not differ substantially depending on whether the group was mixed or non-mixed.

### Ethical considerations

Parents were informed that their participation was voluntary and that all data would be kept confidential. Participants were informed about the study procedure several times, both orally and in writing. Furthermore, all participants were informed to contact the researcher afterwards if they had questions about the study or if they needed someone to talk to about issues raised afterwards.

Ethical approval for the study was obtained from the Regional Ethical Review Board in Uppsala, Sweden (dnr 2013/296).

## Results

The main category that emerged from the FGDs on Somali-born parents’ experiences and needs for parenting support in Sweden was *Parenthood in transition.* The participants described a process of being a parent in the home country compared with the challenges and needs of being a parent in the new country. This parenthood in transition involved cultural and social challenges but was also described as an opportunity for improvement.

**Fig. 1:**
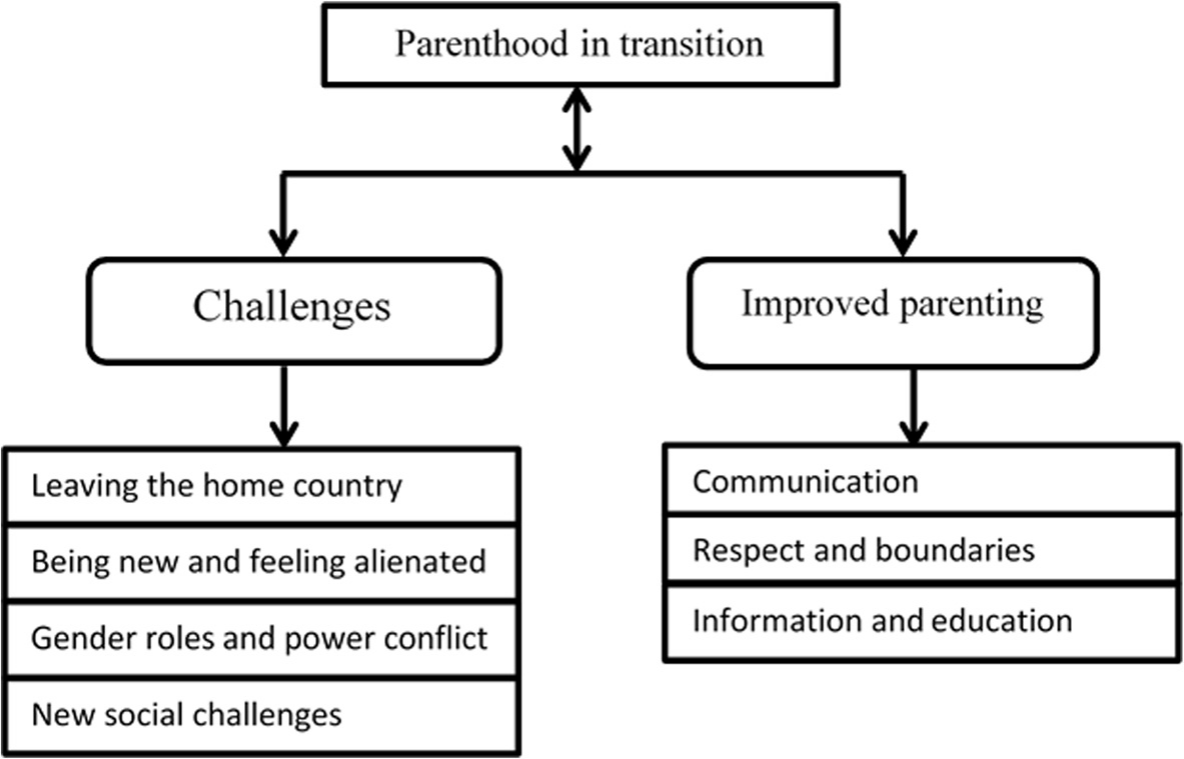
Categories of Somali parents’ experiences and needs for their parenting

Two generic categories were related to parenthood in transition: the *challenges* of leaving the home country and being new and feeling alienated in the new country. The other related category was *Improved parenting* and illustrates what the Somali parents strove for and what they described as needs to achieve better parenting. The process of parenting in transition is illustrated in Fig. 1. In the following sections we present the generic categories and the related sub-categories identified in this process.

### Challenges

The challenges described by the participants included leaving the home country. There had been a forced migration due to the civil war and conflict in the home country, and they had experienced, and continued experiencing, loss of everything that was familiar. Being new and a stranger was a challenge parents faced when they came to the new country, reflecting lack of contextual knowledge and language. The participants felt that they were facing new social challenges in the new country, challenges caused by the stressful living conditions.

#### Leaving the home country

In the interviews, the participants described three challenges related to *leaving the home country*: the effects of the war, difficulties living apart from the family, and the lack of social network support. Their lives had been interrupted and they had been forced to leave everything behind – their houses, families and jobs. Furthermore, they described how families had had to be separated and how chaos and killing had become part of their and their children’s daily lives. Participants reported that most children in Somalia had witnessed people being killed, which had had an impact on both their children’s and their own mental health. Some parents emphasized the need for the Swedish authorities to acknowledge their and their children’s needs for special care including mental health treatment. Some participants mentioned how they had perceived themselves as healthy when they were in their home country, and now they realized they were not. One father said:

The father and mother, when they were in their country they were mentally healthy, as they believed, but when they came here they realized that they were insane, and every person that gets out of that life realizes that he/she is insane … later when I came here, for the first 6 months I wasn’t normal. My brother used to bring me sleeping drugs, I would take that and sleep, then later on I became fine.

Furthermore, the participants were concerned and worried about the relatives they had left behind in Somalia and about the conditions they lived under in Somalia, as reported in the news. These worries had an effect on their and their children’s mental health.

Finally, participants experienced lack of social network support as a result of leaving Somalia. Some said they thought that being a parent in Somalia was easier than being a parent in Sweden. They described the social network in Somalia as helpful. For example, the social network provided babysitting and child-minding support. One father explained:

In Somalia the child might be playing outside .... my neighbour might talk to him if he is doing something wrong. Even the teacher or anyone who sees [him] can talk to the kid if he is doing wrong. By contrast, here in Sweden it’s only you who will care for the kid, so I think that contributes to why it’s hard to be a parent in Sweden.

Participants said that in Somalia, relatives, friends and neighbours had the same role as parents, especially when the parents were not around. Hence, the participants reported that being in a different environment where this was not the case created a sense of loneliness both for themselves and for their children.

#### Being new and feeling alienated

The challenges that resulted from being new and feeling alienated in a country were related to lack of knowledge about the new country, lack of understanding and communication between parents and children.

Lack of knowledge about the culture, the legal system, the language and the new environment all presented challenges for the parents. They relied on other Somali parents who had come to Sweden before them, to act as source of information for them. However, the information received through other parents could sometimes be misleading. Participants reported fear that their children might be separated from them because they were not “good enough” as parents, the parents withheld their health problems or other problems they might have with their children from the social services.

Moreover, most parents described how conflicts most often occurred with older children, 10 years and older. Because of long separation due to the war and because families had fled separately, parents and children, now reunited, often had different expectations of each other. They had difficulty understanding each other and conflicts seemed unavoidable. In addition, lack of communication between parents and their children was another reason for conflict. Somali parents often felt that Swedish parents had better communication with their children than they themselves did. One mother said:

I came into the pharmacy. There was a Swedish father and his daughter together, she wanted to press the queue number, but the father had already pressed it and so the daughter became angry and shouted. He kneeled down and apologized for almost 5 minutes…. We don’t do that.

#### Gender roles and power conflict

One challenge that all parents in the focus group discussed was the role changes and power conflicts between parents and between parents and children that occurred in the new country. The role change between parents and children comprised the challenges occurring when the children adapted to the culture, system and language of the new country faster compared with their parents. Parents felt that their children were sometimes their link for information and sometimes the children misinterpreted, or misled their parents. Consequently, this role change led to power conflicts. For instance, children used their power to force their parents to give them what they wanted. This created a sense of powerlessness in parents. One father explained:

When older kids and parents come together in Sweden, and a kid wants some money, like 5 000 Swedish kroner, he says it’s his money. And the parent can’t refuse because he will say, “If you don’t give me the money, I will tell the social services that you beat me.”

Several participants said that in Somalia the parents were responsible, and made decisions, for their children, while in Sweden the responsibilities were shared with the authorities. As a result, some children believed that they had their own welfare money. The parents’ dilemma was that they could not refuse whatever the child asked as the parents believed that the society would believe the child rather than the parents. This created a power conflict between the parents and the child, in which parents felt powerless and unsupported.

In Somalia, mothers and fathers had clear roles, with most mothers being housewives and fathers being the breadwinner. This began to change during the civil war when women started to work outside the home. However, the mothers had help from relatives and others with the household chores and other domestic responsibilities. In Sweden both parents had to work or go to school and they saw this as challenging their relationship. One father said:

The conflicts between husband and wife started during the civil war when women started to work; before that, when the husband comes home and he is not in a good mood, the wife gives him the service he requires, like feeding him, and bathing him till he cools down … . So who now cools down the other? The woman works, the man works, they both take masculine responsibility. They come home and they’re all exhausted – the husband, the wife and the children.

However, some participants reported that some mothers became aware of having more power in Sweden and they used this to solve the couple’s problems. For instance, some mothers threatened their husbands they would leave or else involve the social worker or the police in their conflicts. One male participant described this as follows: “… The mother is showing that she has more power than the father, and the father starts ignoring the children so as to take revenge on the mother, and then the children suffer and will have psychological problems.”

Male and female participants agreed that the power conflict between parents had an impact on the child’s mental health, and that a positive relationship between parents is crucial for children’s health and performance in school.

#### New social challenges

The new social challenges were described as stressful living conditions that emerged in the transition process. There were challenges for the children in school and in society.

Participants experienced stressful living conditions in the Swedish society. They found that everything was scheduled in Sweden and everyone was expected to adhere to this scheduling of time, which was quite different from life in Somalia. Consequently, this created anxiety and parents found it hard to keep up with having children in kindergarten/school and getting them to school on time. The mothers had the biggest struggle, as they were often on their own with the children. Some were single mothers with many children. They reported that the stress at the beginning of their life in Sweden had been difficult and had been a burden, especially for themselves and their children. Living cramped up in a small flat and coping with the new experience of really cold weather had made the family stay indoors and had led to frustration and conflicts.

Moreover, parents reported that their children were also facing many challenges in the new society. There were children who hadn’t attended school in Somalia. They lacked the social skills that Swedish preschool children had. Parents felt that their children had difficulty adjusting to the Swedish school. Language issues prevented the parents from helping their children. They feared that as a result, their children ran the risk of getting into “bad company” and/or losing interest in their schoolwork. Furthermore, participants reported that there were Somali children who were registered with a wrong biological age, either having been put down as too young or having been registered as too old by their parents. Apparently some children were brought into the country as so-called “11 or 12-year-olds” when in reality they were 16 years or older. When a child ended up in a class where his or her classmates were younger than him or her this created mental distress for the child.

The participants emphasized that school staff needed to know more about and be more sensitive to the Somali children’s educational and cultural background as well as the parents’ educational background. As Somali-born parents, they experienced racism and discrimination in different contexts in Sweden, e.g. at their children’s kindergarten and from school staff. For instance, they described that their children were not treated equally with other children in school or kindergarten. Several of the parents had a feeling that some of the staff in school and in kindergarten kept a constant eye on their children. In addition, they described how the school staff acted suspicious of the parents and sometimes questioned their children on what happened at home. Some mothers feared that this special focus could result in the child telling lies just to get attention. One mother related how two members of staff at the kindergarten were irrationally suspicious when one girl in the kindergarten was quiet and was not playing with other children.

The other two ladies [teachers] are talking about the girl, “What is wrong with her?”, “What happened to the girl?”, “Has something happened at home …?” Then I told them, “Perhaps she’s caught a cold, and it’s not yet showing … .” Then the next day the girl got sick.

### Improved parenting

Aside from the cultural challenges encountered in the process of parenthood in transition, improved parenting emerged as a generic category. Many parents said that coming to the new country had made them rethink their roles as parents and adopt a new style of parenting. In order for parents to become more self-confident in their parenting, participants suggested that they wanted to improve their *communication* with each other as well as the communication with their children, developing mutual *respect* between themselves and their children, and, further, setting *boundaries*. Similarly, they needed support from the authorities, such as relevant *information* and *education* about the society and their rights as parents and about how to parent in the new society.

#### Communication

Good communication, both within the couple and between the parents and their children, was described as being of major importance for successful parenting. Therefore, parents emphasized that they needed to collaborate with and support each other. Parents emphasized the importance of becoming friends with and supportive of their children and the increased need for communicating with their children and spending time with them. One father said, “… children are mostly expecting their parents to listen to them, understand what they are talking about, and respect their opinions and ideas.”

Parents believed that their responsibility as parents was to give their children confidence in who they were, pride in their heritage, and support in becoming part of the new society. In comparison with the traditional upbringing in Somalia, where physical punishment was normal, participants suggested that parents could implement other strategies such as taking away what the child most treasured, coming to agreements with the child and sharing responsibilities and decisions with them.

#### Respect and boundaries

The participants talked about that their children had no respect for them and that they ignored their decisions and requests. Parents thought that this was a result of children in Sweden having a lot of freedom. As a result of that freedom, parents found that their children did not listen to them and they (the parents) could not set any boundaries. The participants also said they had lost their authority as parents and, further, that children’s demands and desires had increased.

All participants said they wanted their children to respect and listen to both them and other adults. As parents, they saw their duty as teaching their children responsibilities, teaching them how to behave in society and also setting boundaries for their children. They said that parents should also affirm their children and respect them.

Parents strove to be role models for their children in order to retain their parental authority. The key to being a role model, was, according to the participants, to learn the language and try to develop their own career. Some parents, however, emphasized that they had sacrificed their career in order for their children to get a better life in Sweden. One mother said,

When I came here I had my four kids, and their father wasn’t around. I had completed high school by then and was expecting to go to university to study economics. I didn’t even get the time to read the language books when I arrived here. I had to work hard to be a father and a mother and a friend to my children.

#### Information and education

The participants had experienced lack of information about systems in the new country. They felt that the information from the integration programme was inaccurate. As mentioned previously, their primary source of information was Somali-born parents who had arrived in Sweden before them. They lacked knowledge about social regulations and there was a clear need for correct and relevant information about their rights as parents. One father commented, “Parents need to know their responsibility and that they can set boundaries for their children, because they were just told not to use physical punishment. But they were never told other alternatives.”

Participants said that better parenting support would improve their parenting. For instance, they requested information on positive parenting and wanted to find ways to parent other than authoritatively. Also, they wanted more information on parenting systems and support in the new country. Parents compared the upbringing their children had had in Somalia with that they were given in Sweden. They said that children’s rights were safeguarded in Sweden, which they thought was a good thing. However, this led to the children’s independence, which resulted in feelings of failure in the parents when, as they felt, their children did not listen to their advice.

Participants said they felt a key to improving their parenting was understanding the culture of and systems in place in the new country. However, they felt it was important to create mutual understanding of each other’s cultures and have a “cultural broker” – a person who could bridge the gap between the services in the municipality and the Somali group. Parents also emphasized the importance of collaboration between them and the school, and also between the school and their children. They thought this was crucial for their children to integrate into the school system. Finally, participants mentioned that the children needed to be taught about parents’ and children’s rights and to understand the rules and the society they were living in now.

Participants had suggestions on how to tailor and deliver such parenting intervention. They stressed the importance of a facilitator who is culturally competent both in the Somali and Swedish culture as well as delivering the parenting intervention in Somali language. When asked if they preferred separate or mixed sessions a majority of the parents preferred a mixed group of both mothers and fathers. One mother said; *“both are parents, it’s better to go together because you will benefit something from one another”*. Participants also recommended that the parenting intervention should be incorporated in the language schools which all individuals who come to Sweden as immigrant attend.

## Discussion

The present study was initiated to collect experience-based knowledge about Somalis’ parenting in Sweden and the need for support they require in relation to this. Our study shows that parenting is a process of transition particularly when immigrating to and living in a host country. Within this process, the impact of forced migration on the participants and their families is huge. Participants highlighted challenges of, and obstacles to, parenthood in transition and presented their ideas on what could contribute to successful parenthood in the new home country.

### Barriers to the parenting transition

Our results highlight a number of barriers to a successful parenting transition, such as difficulties in understanding the system in and culture of the new country, lack of social network support, and lack of communication and understanding between parents and children. These findings may be illustrated the framework of Ochocka & Janzen [31] for understanding immigrant parenting. On a parenting orientation and parenting style level, parents were driven and shaped by their cultural beliefs, family values and how they had brought up their children in home country. In the host country context, participants experienced barriers to a successful parenting transition, such as perception of lack of parenting skills in the new country and lack of knowledge about parents’ rights, which is also in line with other studies [2, 19–21, 30]. One particular barrier that our study identified to successful parenting transition was the tension between the parents, and between the parents and their children. The tension was related to their roles and power conflict. In accordance to the Ochocka & Janzen framework [31], parents start to be conscious of differences between their own cultural orientations and parenting styles and those of the host country, in the host country context. It is in this process that parents start to re-evaluate and modify their parenting beliefs and strategies.

Another barrier to successful parenting transition was loss of the extended family. It has previously been reported that the extended family, neighbours and close friends play the role of mediating between the married couples; they also co-share the responsibility of parenting [2, 22]. These challenges might not be unique for Somali-born parents leaving their home country and fleeing to another country. However, for Somali-born parents, these challenges created stress relating to systems in the host country and fear of losing their children, which caused them not to seek support from the authorities.

Even with knowledge of the country and its systems, parents need support to re-orientate themselves and reshape their approach to parenting in order to achieve a successful parenting transition. Unlike previous results, the participants in our study expressed the desire to re-orientate themselves and modify their parenting and not to continue parenting in line with the traditional upbringing in their home country [2, 19–21, 30]. The participants did want their children to obey them, but they also requested positive parenting guidance helping them to modify their parenting styles. It is on this level of the framework, *Modifications of Orientations and Styles* [29], that parents start to realize they need to modify their parenting style.

### Successful parenting transition

Unlike previous studies exploring immigrant parents’ experiences in their new home country, this study contributes the broader perspective that Somali-born parents see not only challenges but also opportunities for themselves to improve their parenting skills in the new country, despite insufficient support. According to the framework of Ochocka & Janzen [31], in order for immigrant parents to adjust and modify their cultural orientations and parenting styles, they need support in adjusting to and integrating into the new society. Furthermore, they also need to find new ways of parenting. Participants in our study had several solutions for how to adjust their parenting. A prerequisite, according to them, was to learn how to better communicate with their children and move from an authoritarian to a more permissive parenting style, as well as to get support from the authorities to endorse their parenting and empower them as parents. As long as they felt the authorities were not supporting them, they were unable to set boundaries for their children. According to the final component of the framework [29], parents need support to understand the context they are living in, how the society works and how to integrate into the society, to be supported in the process of parenthood transition in the new host country. To empower parents is to inform them about their rights as parents, as well as their children’s rights vis-a-vis them.

Based on the results of the present study, our recommendation is to offer parenting interventions that focuses on parent–child relationship, as our participants desired to adapt another parenting style as well as improve the relationship and communication with their children. Another suggestion is to give parents knowledge on legal rights and obligations as parents in the host country. Parents in our study stressed a fear for social services which caused a tension between parents and children, this fear also undermined the parent–child relationship. A gender mixed parenting intervention was preferred by the participants and the facilitator to be Somali-born with knowledge of the host country.

### Strengths and limitations

This study has some strengths and weaknesses that should be outlined. One of the strengths is that the FGDs were conducted in the participants’ mother tongue and without an interpreter. When a study uses interpreters the meaning can get lost in translations. In our study, the first author, F.O., is both bilingual and bicultural and has previously conducted FGDs. She has implicit knowledge of both cultures, which can be seen as both a weakness and a strength. She came to Sweden as a refugee, which may have influenced her participation in the FGDs. It may also have prevented her from asking certain questions of participants, as well as affecting how open coding was performed. To overcome bias, the moderator and observer compared notes and discussed the outcome and how they both perceived the FGD and certain issues that aroused. Furthermore, all authors have maintained a constant dialogue during the analyses and the writing of the study, so as to enhance credibility.

This study was conducted in a small group of Somali-born parents living in one city in Sweden; therefore the results cannot be generalized to all Somali immigrants. Another limitation might be that parents who did participate in the focus group had extensive experience and interest which might have influenced the content of the focus group discussion. The inclusions criteria excluded some parents who were interested to participate in the FGD but hadn’t been living long enough in Sweden with their children. Nothing indicates that their experiences differ significantly from the parents who participated in the FGDs, however, the parents who declined participation may be important to also gather knowledge and experiences from.

## Conclusions

Somali-born parents’ experiences in this study were shaped by the challenges and opportunities they encountered in the new country. The participants expressed the desire to move forward and focus on their future and the future of their children. In order to have a successful parenting transition, they needed parenting support. Our findings support that Somali parents coming from a war-torn country have different needs of support to assume their parent role in Sweden. This support spans two interconnecting areas; one is about how society works, legal rights and obligations as parents and the social expectations placed on parenthood in the new culture. The second is about parental style and the ability to communicate and bond with the child. Furthermore, our study suggests that professionals in social services and in schools should incorporate their parenting support and communication training for immigrant parents throughout their children’s childhood.

### Further research

Although this study provides some knowledge about the need for culturally sensitive parenting support programmes, we do not know the children’s experiences and how immigration affects them. Future research should explore how Somali-born children manage their lives in the new host country and what their needs are in order to promote their mental health. Furthermore, future research should develop, implement and evaluate culturally sensitive parenting support programmes.
